# Influence of Stimulus Size on Simultaneous Chromatic Induction

**DOI:** 10.3389/fpsyg.2022.818149

**Published:** 2022-01-24

**Authors:** Tama Kanematsu, Kowa Koida

**Affiliations:** ^1^Department of Computer Science and Engineering, Toyohashi University of Technology, Toyohashi, Japan; ^2^Japan Society for the Promotion of Science, Tokyo, Japan; ^3^Electronics-Inspired Interdisciplinary Research Institute (EIIRIS), Toyohashi University of Technology, Toyohashi, Japan

**Keywords:** psychophysics, color vision, spatial vision, illusion, eye optics

## Abstract

Chromatic induction is a major contextual effect of color appearance. Patterned backgrounds are known to induce strong chromatic induction effects. However, it has not been clarified whether the spatial extent of the chromatic surrounding induces a chromatic contrast or assimilation effects. In this study, we examined the influence of the width of a center line and its flanking white contour on the color appearance when the line was surrounded by chromatic backgrounds. A strong color shift was observed when the center line was flanked by white contours with the L/M- and S-cone chromatic backgrounds. There was a difference between the optimal widths of the center line and the contour for the shift in color appearance for the L/M-cone chromaticity (0.9 and 1.1–1.7 min, respectively) and the S-cone chromaticity (8.2–17.5 and 0.9–2.5 min, respectively). The optimal width of the center line for the L/M-cone was finer than the resolution-limit width of the chromatic contrast sensitivity and coarser than that of the luminance contrast sensitivity. Thus, the color appearance of the center line could be obtained by integrating broad chromatic information and fine luminance details. Due to blurring and chromatic aberrations, the simulated artifact was large for the darker center line and S-cone background, thus suggesting that the artifact could explain the luminance dependency of the induction along the S-cone chromaticity. Moreover, the findings of this study reveal that the dominant factor of the color shift is neural instead of optical.

## Introduction

Human visual perception is influenced by spatial contexts such as chromatic surroundings, luminance, and size. The spatial contexts can induce remarkable visual illusions. Thus, various artists and designers precisely control chromaticity and shapes to produce visual effects. The influence of the surrounding chromaticity (chromatic induction) on the centered area can be classified as assimilation and contrast depending on the direction of changes in the color appearance. The first is the simultaneous chromatic assimilation that induces a color from the surrounding area into the center and biases the color appearance at the central region toward the surrounding chromaticity. This assimilation is a type of spatial averaging that contributes to reliable chromatic signal detection. The second is the simultaneous chromatic contrast that induces complementary colors from the surroundings and biases the color appearance of the center away from the surrounding chromaticity. The chromatic contrast process facilitates the detection of low-contrast objects and contributes to color constancy (Lotto and Purves, 2000; Hurlbert and Wolf, 2004). To fully understand these chromatic inductions, it is necessary to assess the contributions of optical and neural factors to various stimulus sizes and luminance contrasts, given that both factors generally lead to similar shifts in color appearance.

**FIGURE 1:**
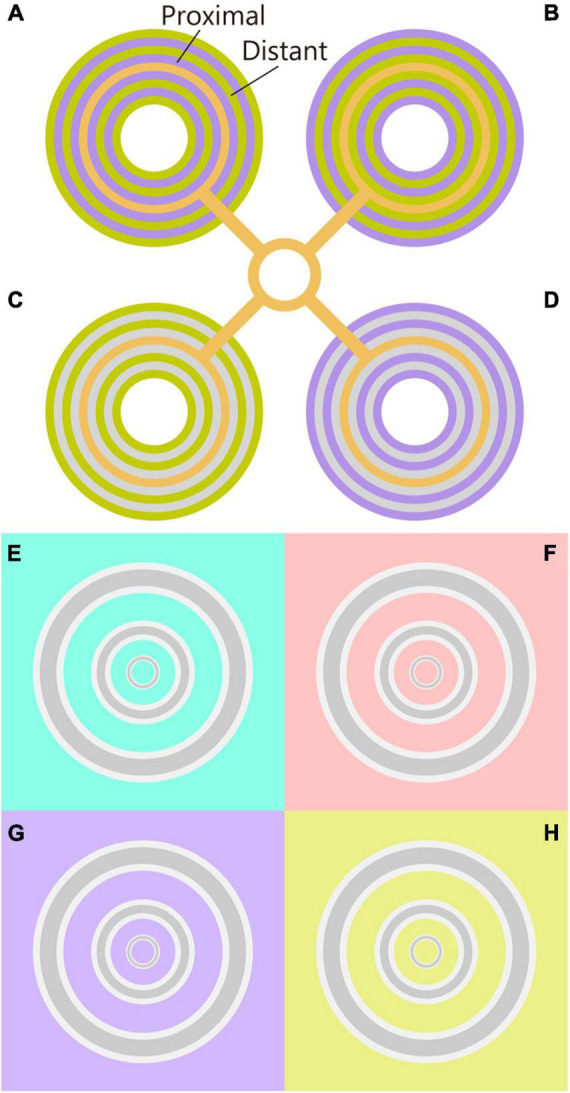
Chromatic induction demos. **(A,B)** The original Monnier–Shevell illusion. Color appearance of center rings were different in panels **(A,B)**, although their physical chromaticity was the same. The chromaticity of the proximal and distant rings was inverted in panels **(A,B)**. The color appearance of the center ring was similar to the proximal color. Thus, the color appearance can be interpreted as the assimilation effect. **(C,D)** Chromatic contrast effect of the Monnier–Shevell illusion. Given that the chromaticity of proximal rings was achromatic, the assimilation effect from proximal rings was the same for panels **(C,D)**. Nevertheless, the color appearance of the center rings was different in panels **(C,D)**. Thus, the color appearance can be interpreted as the contrast effect from the distant rings. **(E–H)** The chromatic induction demos (Kanematsu and Koida, 2020). The color appearance of gray rings differed among the chromaticities of the backgrounds **(E–H)** although the physical chromaticity of the rings was the same. Concentric gray rings were flanked by white contours. The innermost ring appeared reddish on the cyan background **(E)**, and cyan on the red background **(F)**. Moreover, all the rings appeared yellowish on the violet background **(G)** and bluish on the yellow background **(H)**. Note: please adjust the viewing distance for the effective illusion, and maintain a distance from the figure to maximize effectiveness.

Monnier and Shevell (2003) reported that surrounding rings alternating between two chromaticities produces a strong shift in the color appearance of the centered ring. This chromatic induction was interpreted as an additive integration from the chromatic assimilation effect of the proximal rings and the chromatic contrast effect of the distant rings (Monnier and Shevell, 2003, 2004; Mély et al., 2018). For example, when the proximal and distant rings were violet and lime-yellow, respectively, the color of the centered ring was strongly perceived as bluish (the orange line appears pinkish in Figure 1A). Switching the chromaticity between the proximal and distant rings induced a greenish color to the test ring (the orange line appeared yellowish in Figure 1B). Similar color shifts were observed even when the proximal ring was achromatic, which was due to the contrast effect of the distant rings (Figures 1C,D; Monnier and Shevell, 2004). However, it is known that the alternating surroundings are not essential for the color shift, given that single proximal and distant surroundings induce a color shift (Mély et al., 2018). These simplifications indicate that the proximal and distant surroundings are important factors in chromatic induction.

Similar to ordinary chromatic induction (Kirschmann, 1891; Kinney, 1962; Bergström and Derefeldt, 1975; Bergström et al., 1978; Fach and Sharpe, 1986; De Weert and Spillmann, 1995; Pinna et al., 2001; Smith et al., 2001; Cao and Shevell, 2005; Gordon and Shapley, 2006), the Monnier–Shevell illusion is dependent on the ring width (Shevell and Monnier, 2005) and luminance contrast (Cerda-Company et al., 2018). The width dependency of the Monnier–Shevell illusion was investigated for the S-cone chromaticity, given that the illusory effect was prominent for S-cone chromaticity, although the effect was observed for the L/M-cone chromaticities (McCamy, 2003; Lin et al., 2010; Cerda-Company et al., 2018). The neural mechanism of the Monnier–Shevell illusion is based on the excitatory-center and the inhibitory-surrounding organization receiving inputs from the S cone (Monnier and Shevell, 2004; Mély et al., 2018). It was conjectured that the significant effect does not occur only for the S-cone chromaticity, given that the center-surround organization in V1 occurred for the L/M-cone chromaticity (Lennie et al., 1990; Johnson et al., 2001, 2008; Chatterjee and Callaway, 2003; Conway and Livingstone, 2006; Brouwer and Heeger, 2009). This physiological evidence questions whether previous reports of weak illusional effects for L/M-cone chromaticities (Cerda-Company et al., 2018) could be due to the non-optimal, large width of the stimuli.

We previously reported that a gray line with white flanking contours appeared reddish on a cyan background, which is similar to an L/M-cone chromaticity (Figure 1E; Kanematsu and Koida, 2020). The observed color shift was larger than the color shift of the line without contours; thus, the chromatic contrast effect was enhanced by the contours. These contours and chromatic backgrounds correspond to the proximal and distant surroundings of the Monnier–Shevell illusion. A thin gray line was found to induce a large color shift; however, we did not examine how the color shift changed with respect to the line width.

Three points remain quantitatively unknown: (1) the optimal width for the illusion modulated along the L/M-cone, (2) the difference in the optimal width when compared with the S-cone, and (3) the influence of optical artifacts on the width and luminance dependency. In this study, we performed a psychophysical experiment by varying the width of a center line, the width of a flanking contour, and the luminance levels of the center line to quantitatively evaluate the following: whether the color appearance changes with respect to the width, and whether the optimal width changes with respect to the surrounding chromaticity. Moreover, we estimated artificial chromatic shifts due to the human ocular optics, and participants were asked to comment on the influence of the optical artifacts on the perceptual color shifts. This enabled us to isolate the optical factors from neural factors.

In this manuscript, we report that the color shift is evident for thin lines when the surrounding has an L/M-cone chromaticity. On a cyan background (-L + M), the thinnest ring on the center appeared reddish, and the thicker rings appeared gray (Figure 1E). On the red background (L - M), the thinnest ring on the center appeared cyan (Figure 1F). On the violet background [S - (L + M)], all the rings appeared yellowish (Figure 1G). Finally, on the yellow background [-S + (L + M)], all the rings appeared bluish (Figure 1H). All the rings were initially and gray, and the flanking contours were white.

## Materials and Methods

### Participants

Seven participants (six males and one female; ages 23–44) with normal or corrected-to-normal visual acuity participated in the experiments. Normal trichromacy was assessed using the Ishihara color plates. The experimental procedure was approved by the Committee for Human Research at Toyohashi University of Technology in accordance with the ethical guidelines outlined in the Declaration of Helsinki. The experiment was conducted in accordance with the approved protocol. Each participant provided informed consent after receiving the procedural details.

### Apparatus

This experiment was performed using a monitor (UP2516D, Dell; 2,560 × 1,440 pixels, Adobe RGB monitor), equipment, script environment (Psychtoolbox 3.0.15, Brainard, 1997 on Matlab R2018a, Mathworks), and calibration with a chroma meter (CS-200, Konica Minolta, Japan), which were the same as those employed in a previous study (Kanematsu and Koida, 2020).

### Stimuli

The color chromaticity of the stimuli was defined by the MacLeod–Boynton (MB) chromaticity coordinates (MacLeod and Boynton, 1979) using the Smith and Pokorny cone fundamentals (Smith and Pokorny, 1975). The spectral intensity of the monitor was measured using an illuminance spectrophotometer (CL-500A, Konica Minolta, Japan) to obtain the cone excitation. Two axes *l* and *s*, the relative activation of the L- and M-cones [*l* = L/(L + M)], and the activation of the S-cone [*s* = S/(L + M)] in an equiluminant plane were considered. The unit of *s* is arbitrary, which was normalized in this study to 1.0 for equal-energy white.

**FIGURE 2:**
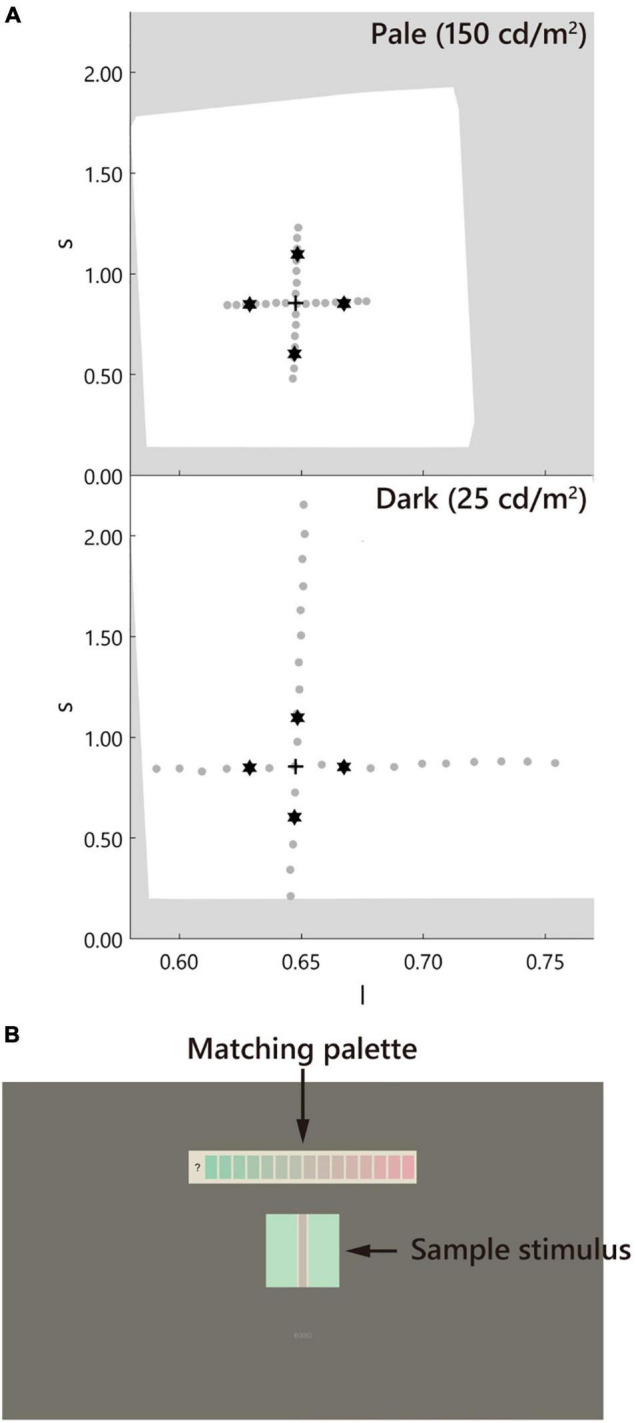
Chromaticity of the stimuli and the screen design. **(A)** The stimulus was defined in the MacLeod–Boynton (MB) chromaticity space. The stars, cross, and gray dots indicate the inducer, D65 of the neutral gray, and the matching palettes, respectively. The white background indicates the gamut of the monitor. The upper and lower panels correspond to the pale (150 cd/m^2^) and dark (25 cd/m^2^) test line conditions, respectively. Note: the luminance of the inducer was maintained at 190 cd/m^2^. **(B)** The screen. The stimulus was placed at the center of the screen. The matching palette was placed above the stimulus.

**FIGURE 3:**
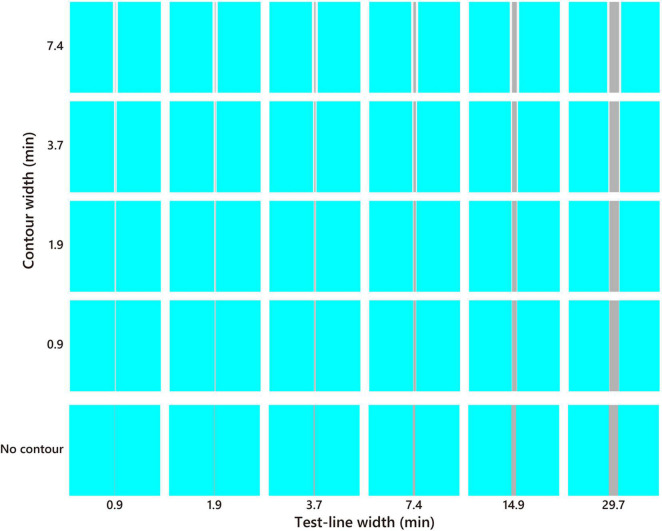
Stimulus example of the cyan inducer. The test line increased in thickness toward the right, and the white contour increased in thickness toward the top. The no-contour condition is shown at the bottom. The color and brightness were optimized for presentation.

The sample stimulus was a vertical straight line (*test line*) tinted in pale or dark gray (D65, *l* = 0.66, *s* = 0.87, CIE *x* = 0.313, *y* = 0.329, luminance = 25, 150 cd/m^2^; Figure 2). It is known that a straight line induces a similar illusory effect as the Monnier–Shevell illusion (McCamy, 2003). A straight line is convenient for displaying stimuli with an exact width per pixel. The widths of the test lines were 0.9, 1.9, 3.7, 7.4, 14.9, and 29.7 min (1, 2, 4, 8, 16, and 32 pixels; Figure 3). The test line was placed at the center of an inducer area (4.8° = 312 or 311 pixels square; Figure 2B). The inducer chromaticity was selected along the cardinal axis of the MB chromaticity coordinates (Figure 2A). Chromaticities of the inducers were cyan (*l* = 0.64, *s* = 0.87, CIE *x* = 0.278, *y* = 0.329), red (*l* = 0.68, *s* = 0.87, CIE *x* = 0.340, *y* = 0.330), violet (*l* = 0.66, *s* = 1.12, CIE *x* = 0.297, *y* = 0.291), and yellow (*l* = 0.66, *s* = 0.61, CIE *x* = 0.334, *y* = 0.380). The luminance of the inducer area was 190 cd/m^2^. Two stimuli conditions were used (Figure 3): a white-contour condition with a flanking white contour (D65, 210 cd/m^2^) and a no-contour condition without a flanking contour. The widths of the contours were 0.9, 1.9, 3.7, and 7.4 min (1, 2, 4, and 8 pixels, respectively; Figure 3).

A matching palette made of horizontally aligned chromatic rectangles (0.8° × 1.6°) with a gap of 0.2° was used. This palette was placed above the stimulus with a gap of 2.0° (130 pixels). The luminance of the rectangles and the test line was the same. The chromaticity of the palette was evenly spaced along the cardinal axes of the MB chromaticity space, including the neutral gray D65 (Figure 2A). The number of rectangles was 15–17 depending on the chromaticity of the inducer. The density of the palette on the MB chromaticity was higher for the pale condition and lower for the dark condition. These ranges and densities were determined based on the participant responses in the preliminary experiment. The rectangles were placed on a white background (D65, 210 cd/m^2^) to maintain the same luminance contrast as that of the test line of the white-contour condition. The screen outside the stimulus and matching palette was painted gray (D65, *l* = 0.66, *s* = 0.87, CIE *x* = 0.313, *y* = 0.329, 50 cd/m^2^, 39.6° × 22.3°).

### Procedure

After the participants adapted to a dimly illuminated room for 5 min, the experiment was initiated. The head of each participant was fixed on a chin rest at a viewing distance of 80 cm from the monitor. The participants were then instructed to select the rectangle from the matching palette that matched the color appearance of the test line (Figure 2B) using the mouse. Fixation was not required. Thereafter, the participants pressed the button to proceed to the subsequent trial. A 300-ms blank screen (D65, 50 cd/m^2^) was shown between each trial. The total number of presented stimuli was 240, including four inducers, two luminance levels, six widths of the test lines, four widths of the white contour, and the no-contour condition. The 240 stimuli were presented in random order. Participants were provided with a break of approximately 4 min after every 80 matchings, and all the matchings were completed after approximately 40 min (including breaks). This experiment was conducted three times for each participant, with a minimum break of 10 min between sets.

### Analysis

The participants were permitted to select the question mark on the left of the palette if no suitable chromaticity was present on the palette or if they were unable to distinguish the test line. Even in the most frequent case, the percentage was less than 10% for each stimulus (Supplementary Figure 1). Therefore, we concluded that the responses to the question had a low impact, and such responses were considered as missing data.

To compare the matching across different chromaticities of the inducers, the matched chromaticity was normalized by the inducer chromaticity based on the metrics of the MB chromaticity. Hereafter, we refer to the *contrast effect*, similar to the metric in a previous study (Cerda-Company et al., 2018), as Δ*E*_*m*_/Δ*E*_*i*_, where Δ*E*_*m*_ is the distance of the matching from D65, and Δ*E*_*i*_ is the distance of the inducer from D65. The contrast effects of the cyan and red inducers are defined by the *l* axis. The matching of the violet and yellow inducers is defined by the *s* axis. A positive contrast effect indicates a color shift away from the inducer, a negative contrast effect indicates a color shift toward the inducer, and zero indicates no color shift.

All analyses were performed using the fitrm and ranova functions (Statistics and Machine Learning Toolbox 12.0, MATLAB R2020b, MathWorks) for N-way analysis of variance (ANOVA) with repeated measures.

To examine the difference in the optimal width, which was distributed in two dimensions between the two groups, we performed a bootstrap test. The optimal width presented in the main analyses was obtained after averaging, smoothing, and then determining the maxima. Here, the optimal width was obtained individually instead, and 21 optimal widths were obtained (seven participants repeated three times). The distance between the two groups was determined by the Euclidean distance between the mean of each group in the metrics of log (min). The null hypothesis was that the optimal widths of the L/M- (including both cyan and red inducers) and S-cone chromaticities (including both violet and yellow inducers) were the same. Based on the null hypothesis, the optimal widths were randomly shuffled across the two groups, and the mean distance was obtained. This re-sampling was performed 2,000 times to obtain the distribution of the mean distance. Subsequently, the distance inducing 95% of the distribution was calculated and compared with the original distance in the experiment. Moreover, the bootstrap test of the luminance levels for each cardinal axis was performed.

## Results

### Optimal Line Width

**FIGURE 4:**
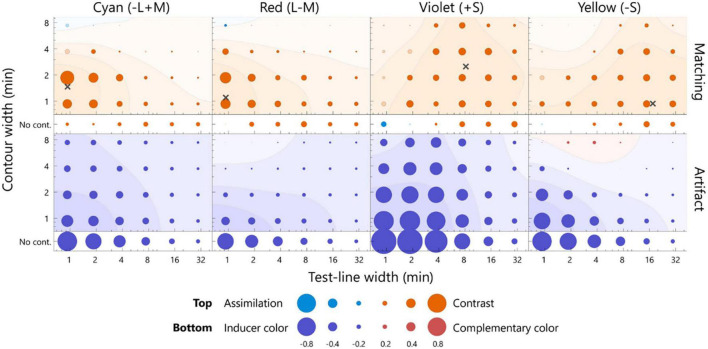
The averaged matching across all the participants **(top row)** and the estimated artifacts **(bottom row)** for the pale test line. The chromaticities of the inducers from left to right was cyan, red, violet, and yellow. The horizontal axis indicates the width of the test line, and the vertical axis indicates the width of the contour. The diameter of each bubble indicates the magnitude of the color shift. The actual values for the diameters are below the axis label. The warm colored bubbles indicate the shift away from the inducer, and the cool colored bubbles indicate the shift toward the inducer. The filled bubbles represent a significant difference from zero (*p* < 0.05, sign test). The color shift was calculated on the *l*-axis for the cyan and red inducer, *s*-axis for the violet and yellow inducer, and normalized by the inducer chromaticity (see section “Analysis”). A contour map represents smoothed data based on a two-dimensional Gaussian filter [sigma = log(1.5) for both the horizontal and vertical axes]. The contour map was normalized with the largest positive value and visualized with 25% increments. The red and blue surfaces indicate a shift away from the inducer and toward the inducer, respectively. The points of the widths inducing the largest color shift were estimated from smoothed data, as indicated by the cross marks. The actual values of the matching and artifact were shown in Supplementary Figure 2.

The color appearance of the pale test line is shown in Figure 4. For the cyan inducer, the largest shift toward red was observed at 0.9 min of the test line and 1.9 min of the contour. The observed contrast effect was 0.59, which significantly deviated from zero (Figure 4, *p* < 0.05, sign test). This contrast effect was significantly larger than that of the no-contour condition for the same width of the test line (Supplementary Figure 2, *p* < 0.05, sign test), thus indicating that the white contour induced a strong simultaneous chromatic contrast. The contrast effect gradually faded when the width deviated from the optimal level [*p* < 0.05, *F*(5, 95) = 6.87; width of test line, *p* < 0.05, *F*(3, 57) = 34.44; contour width, 2-way repeated measures ANOVA; see Supplementary Table 1 for complete statistics]. The no-contour condition induced a similar contrast effect for all the widths [*p* > 0.05, *F*(5, 100) = 1.80; width of test line, one-way ANOVA with repeated measures; see Supplementary Table 2 for complete statistics]. Similar trends were observed for the red inducer. For the violet and yellow inducers, significant chromatic contrast effects and width dependencies were observed, although relatively thicker lines resulted in the most significant effects.

**FIGURE 5:**
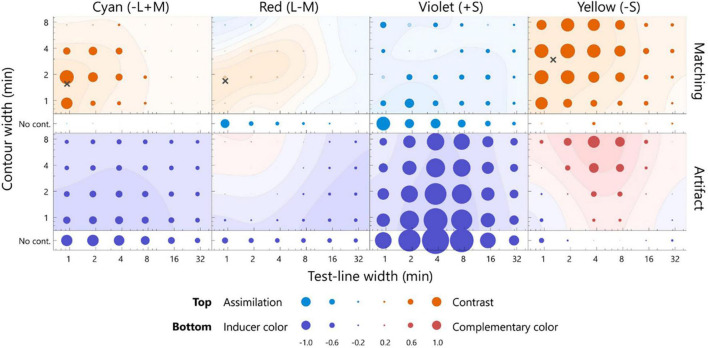
The averaged matching across all participants **(top row)** and the estimated artifacts **(bottom row)** for the dark test line. The format is the same as in Figure 4. Note: the scale of the bubble size is different, as shown in the key below. The points inducing the largest color shift are indicated by cross marks, with the exception of the violet inducer, given that the positive contrast effect was not observed. The actual values of the matching and artifact were shown in Supplementary Figure 3.

To quantitatively estimate the width resulting in the maximum effect of chromatic contrast (hereafter *optimal width*), the matching was interpolated by smoothing (Gaussian SD = 1.5 log unit; see Figure 4 caption). The optimal width was determined to maximize the smoothed surface (the cross markers in Figures 4, 5). The optimal widths of the pale test line for cyan, red, violet, and yellow were 0.9, 0.9, 8.2, and 0.9 min, respectively, and those of the contour for the colors were 1.5, 1.1, 2.5, and 0.9 min, respectively (Figure 4). To compare the optimal widths of the inducers, we calculated the Euclidean distance of the optimal widths of between a pair of the inducers in the two-dimensional stimulus space instead of calculating the differences in the width of the test line and those of the contour separately. The observed optimal widths were significantly different between L/M-cone (the data of the cyan and red inducers were integrated) and S-cone inducers (the data of violet and yellow inducers were integrated) (*p* < 0.05, bootstrap test). Similar conclusion was obtained if significance was tested separately; the optimal width of the test line was different (*p* < 0.01, *t*-test) and the optimal width of the contour was different (*p* < 0.01, *t*-test). The ratios of the optimal width of the S-cone to L/M-cone ranged from 9.1 to 19.4 for the test line and 0.6–2.3 for the contour. Thus, the S-cone had a larger optimal width than the L/M-cone.

The color appearance of the dark test line is shown in Figure 5. For the cyan inducer, the largest shift was observed at 0.9 min for the test line and at 1.9 min for the contour. The observed contrast effect was 1.48, which significantly deviated from zero (*p* < 0.05, sign test). For the same width of the test line, this contrast effect was significantly larger than that in the no-contour condition (Supplementary Figure 3; *p* < 0.05, sign test), thus indicating that the white contour induced a strong simultaneous chromatic contrast. The contrast effect gradually faded when the width deviated from the optimal level [Supplementary Table 3; *p* < 0.05, *F*(5, 100) = 17.16; width of test line, *p* < 0.05, *F*(3, 60) = 11.02; contour width, two-way ANOVA with repeated measures]. Similar width dependencies were observed for the red inducer, although the magnitudes were smaller than those for the pale test line. Consistent chromatic assimilation was observed for the violet inducer. Additionally, the yellow inducer demonstrated a strong simultaneous chromatic contrast.

The optimal widths of the dark test line were 0.9, 0.9, and 1.3 min for the cyan, red, and yellow inducers, respectively, and the those of the contour were 1.6, 1.7, and 2.9 min, respectively (Figure 5). The optimal width for the violet inducer could not be defined because no chromatic-contrast effect was induced. The ratios of the optimal width of the S-cone to L/M-cone were 1.4 for the test line and 1.7–1.8 for the contour. Thus, the optimal width for the S-cone was larger than that for the L/M cone.

We compared the dark and pale conditions. However, the optimal widths for the L/M-cone were not significantly different (for both red and cyan inducers, *p* > 0.05, bootstrap test). It was difficult to compare the results of the violet inducer, given that the sign of the color shift changed. For the yellow inducer, the optimal widths were significantly different (*p* < 0.05, bootstrap test). Moreover, the ratios of the optimal width of the pale to dark test line condition were 13.5 for the test line and 0.3 for the contour.

### Optical Artifacts

The cornea and lens focus images on the retina. Due to the imperfection of human ocular optics, several artifacts emerge on the retinal image. Wavelength-independent aberrations, which are referred to as monochromatic aberrations, blur the image. Wavelength-dependent aberrations, which are referred to as chromatic aberrations, arise from a difference in the refractive index where short-wavelength light is further refracted. There are two types of chromatic aberrations: longitudinal and lateral. Longitudinal aberrations cause a difference in the focus points along an optical axis; thus, short-wavelength light is blurred on the image plane while long-wavelength light is focused. Lateral aberrations occur when long-wavelength light is focused on a farther peripheral position than that of short-wavelength light, thus causing a chromatic fringe at an edge. These artifacts degrade the quality of retinal images, especially the color and spatial details. We used extremely thin stimuli; thus, the artifacts had a significant influence on the image. If the monochromatic aberration causes the light to spread from the inducer to the test line, chromatic shift similar to that of the chromatic assimilation is observed. If the longitudinal chromatic aberration causes the light to spread from the inducer and contour to the test line, a blue chromaticity is induced. To determine whether the color shift observed in the experiment originated from optical or neural factors, it was necessary to predict these optical artifacts quantitatively and compare them with the results of color matching.

Marimont and Wandell (1994) proposed a model for calculating retinal images using human ocular optics. The model integrates a formula representing the wavelength-dependent de-focus (Thibos et al., 1992) and the optical transfer function (OTF), including longitudinal chromatic aberrations (Williams et al., 1994), into the OTF of Hopkin’s eye model (Hopkins, 1955). We applied this model to the stimuli and examined whether the optical artifacts accounted for the color shift observed in the experiment.

The model calculates the retinal image from an input image using ocular optics. The input image was the same as that used in the experiment with a resolution of 0.46 min per pixel. The region of the test line of the output image was averaged in the MB space and then normalized to calculate the contrast effect, similar to the matching experiment. Although aberrations change significantly in both chromaticity and intensity, we determined the artifacts in the MB space to focus on the color shift. The color shift was defined along one of the cardinal axes: the *l* axis for the cyan and red inducers and the *s* axis for the violet and yellow inducer. The pupil diameter used in the calculation was 3.63 mm. This value is appropriate for a background luminance of 50 cd/m^2^ and a screen with a width of 39.6° and height of 22.3° (Stanley and Davies, 1995; Atchison et al., 2011; Watson and Yellott, 2012). All parameters in the model were set to focus on a wavelength of 580 nm (Marimont and Wandell, 1994).

The bottom panels of Figure 4 present the estimated artifacts for the pale test line. The chromaticity of the test line generally shifted toward the inducer chromaticity. This shift was significant for the thin lines. For the yellow inducer, small chromatic shifts away from the inducer chromaticity were observed at the thick contour (7.4 min). For the no-contour condition, consistent shifts toward the inducer chromaticity were observed. The artifact ranged from -0.84 to -0.12, -0.71 to -0.02, -1.09 to -0.17, and -0.83 to 0.16 for the cyan, red, violet, and yellow inducers, respectively, including both the contour and no-contour conditions.

The optical artifacts caused a shift similar to the chromatic assimilation and had an opposite effect compared to that of the matching experiment. We calculated the correlation coefficient between the artifacts and the matching to evaluate similarities in the dependences of the line widths. The correlation coefficients were negative for all the inducers (cyan: *r* = −0.78, *p* < 0.05; red: *r* = −0.83, *p* < 0.05; violet: *r* = −0.19, *p* > 0.05; yellow: *r* = −0.48, *p* < 0.05). Therefore, the optical artifact did not account for the illusory effect, particularly for the cyan and red inducers.

The bottom panels of Figure 5 present the estimated artifacts for the dark test line. The chromaticity of the test line shifted toward the inducer chromaticity for the cyan and violet inducers, and the chromatic shift was maximum at the test line of 3.7 min for both. The chromatic shift for the red inducer was weak; however, it was sufficient, and a shift toward the inducer chromaticity was observed for the thick lines. For the yellow inducer, the chromaticity of the test line generally shifted away from the inducer chromaticity and exhibited a maximum shift at the 3.8-min test line with a thick contour. The artifacts ranged from −1.25 to −0.42, −0.68 to 0.14, −2.90 to −0.81, and −0.58 to 1.29 for the cyan, red, violet, and yellow inducers, respectively, including both the contour and no-contour condition. It should be noted that the bubble scaling differed from that in Figure 4.

The directions of the artificial shifts for the cyan and red inducers were opposite to those of the color shift observed in the matching. On the other hand, for the violet and yellow inducers, the directions of the artificial shifts were similar to those of the matching. Correlation coefficients between the matching and artifacts were significantly negative for the cyan inducer (*r* = −0.51, *p* < 0.05); however, there was no correlation for the red inducer (*r* = 0.14, *p* > 0.05). Therefore, the artifacts did not account for the illusory effect along the L/M-cone chromaticities. The correlation coefficients for the violet and yellow inducers were positive and non-significant (violet: *r* = 0.40, *p* > 0.05; yellow: *r* = 0.37, *p* > 0.05). Thus, we could not exclude the contribution of artifacts to the illusory effect along the S-cone chromaticities.

## Discussion

### Optimal Width and Extent of Inhibitory Surroundings

We found optimal line widths for the color shift. The observed optimal widths from the S-cone inducers were moderate, and those from the L/M-cone inducers were thin. The observed optimal widths and the magnitudes of the color shift were compared with those observed in previous studies, as follows.

Shevell and Monnier (2005) measured the magnitude of the color shift from S-cone-patterned surroundings by independently changing the width of the centered ring and surrounding stripes. The magnitude of the color shift was maximum when the test line was the thinnest (however, tests were conducted for widths of up to 6 min) and the surrounding stripe was 3.3 cpd (the medium frequency they used, Shevell and Monnier, 2005). To compare the abovementioned results with those from this study, the pale test line (-21% luminance) was considered instead of the dark test line (-87% luminance), given that the previous study used a +33% brighter test line stimuli on the patterned background. The results of the present study revealed that the color shift for the violet and yellow inducers was the strongest when the width of the test line was 8.2–7.5 min and that of the contour was 0.9–2.5 min. This optimal width of the test line was slightly wider; however, it was similar to that obtained by Shevell and Monnier. In contrast, the optimal contour width (0.9–2.5 min) was 3.6–10.1 times less than that in the study conducted by Shevell and Monnier (3.3 cpd; 9 min for each stripe).

It should be noted that the optimal width was lower than in previous studies. One possible explanation is the difference in the surrounding patterns. Shevell and Monnier used stripes of rings with alternating S+ (violet) and S- (lime) chromaticities. This alternating surrounding was made of the first proximal (say S+), second proximal (S-), third proximal (S+), and other higher-order proximal. Due to the spatially antagonistic center-surround receptive field, as the neural substrate mediating the color shifts, the extent of the surrounding suppression determines the contrast effect (Mély et al., 2018). If the extent of suppression is larger than that of the second ring, the suppression region is exposed to both the second (S-) and third (S+) chromaticities. The amplitude of the surrounding suppression is consequently weakened because of the elimination of the second and third inducers. To maximize the contrast effect using an alternating stimulus, the second ring should be sufficiently thick to cover the extent of the suppressive surrounding. Given that Shevell and Monnier modulated the stimulus width of the first, second, and third rings simultaneously, the low spatial frequency and thicker second ring was effective for the color shift. In contrast, the stimulus in our study had a uniformly chromatic surrounding, with the exception of the contour; thus, the colors did not cancel each other in the suppressive region. Therefore, the optimal width was less than those obtained in previous studies wherein alternating stimuli were used.

We observed a strong color shift for the L/M-cone when the fine test line and contour were used. Although previous studies revealed that the Monnier–Shevell illusion emerged along the L/M-cone (McCamy, 2003; Lin et al., 2010; Cerda-Company et al., 2018), the optimal width of the illusion for the L/M-cone was not investigated. The line widths used in those previous studies were 9 min (Lin et al., 2010) or 15.5 min (Cerda-Company et al., 2018) and were sufficient for the S-cone. However, it was unclear whether the widths were appropriate for the L/M-cone. The results revealed that the optimal width was 1.7 min for the test line and 1.6–1.7 min for the contour with respect to the L/M-cone. In this study, the optimal widths were 5–9 times smaller than the widths used in previous studies. Thus, the Monnier–Shevell illusion for the L/M-cone could be more significant if a thinner patterned surrounding is used. Cerda-Company et al. (2018) revealed that the magnitude of the effect for the L/M-cone was smaller than that for the S-cone. However, in our current study, the results revealed comparable effects for the L/M-cone and S-cone when the optimal width was used. Given that the stimuli were different in spatial configuration from those used in the study conducted by Shevell and Monnier, further research is required to examine the appearance of a thinner L/M-cone patterned background in the Monnier–Shevell illusion.

The illusory effect of the L/M-cone chromaticities was evident for the contours and test lines with small widths, and the optimal widths for the L/M-cone were smaller than those for the S-cone. To reconcile this discrepancy, we proposed that different spatial resolutions for each cardinal axis led to different optimal widths. It is known that the two cardinal axes originate from different types of cones in the retina. The density of each cone type is not uniform. In particular, the S-cone only accounts for 5% of the population (Roorda and Williams, 1999; Calkins, 2001; Hofer et al., 2005). The densities of the L- and M-cone are 67 and 28%, respectively, on average, although there are large individual differences (Roorda and Williams, 1999; Hofer et al., 2005; Neitz et al., 2020). The midget ganglion cell at the fovea receives the input from a single L or M cone to its central receptive field (Wool et al., 2019) and conveys the fine details of the L/M-cone information. In contrast, the bistratified ganglion cell receives inputs from several S cones; thus, the spatial details of individual S-cone information are lost (Wool et al., 2019). These retinal properties are preserved in the lateral geniculate nucleus (LGN). Thus, the parvo cells of the LGN, which receive inputs from the midget cell, convey the fine details of the L/M-cone information. The konio cells, which receive inputs from the bistratified cell, convey the relatively coarse S-cone information (Irvin et al., 1993; Xu et al., 2002; Tailby et al., 2008). Reflecting this early visual processing, the cells in the cortex respond to higher spatial frequencies of the L/M-modulating stimuli than those in the S-cone (Wandell and Silverstein, 2003; Solomon et al., 2004; Johnson et al., 2010). Psychophysical experiments revealed that observers can detect higher spatial stimuli that modulate the L/M-cone chromaticity than those that modulate the S-cone chromaticity (Mullen, 1985; Webster et al., 1990; McKeefry et al., 2001; Mullen and Kingdom, 2002; Barboni et al., 2013). This suggests that the spatial resolution of color vision is higher for the L/M-cone chromaticities than for the S-cone chromaticities. Assuming that the center-surround organization is the neural basis of the Monnier–Shevell illusion (Monnier and Shevell, 2004; Mély et al., 2018), the fact that the optimal width for the L/M-cone is smaller than that for the S-cone chromaticity is in accordance with the property of color vision from the retina to perception.

### Integration of Chromatic and Luminance Signals

The optimal width for the L/M-cone chromaticities was 0.9 min (33.3 cpd) for the test line, and it was equivalent to a resolution level of one cone photoreceptor (approximately 1 min at fovea, Williams and Coletta, 1987). This width is nearly equal to the resolution limit for the luminance grating (the critical fusion frequency of 32–36 cpd, which corresponds to 0.9–0.8 min sampling, Mullen, 1985). The stimuli could be detected by the luminance channel. However, the critical fusion frequency of the L/M-cone chromatic stimuli is 11–12 cpd (Mullen, 1985); hence, the test line and the contour were hardly detectable solely by the chromatic channel. Given that the test line and contour had the same chromaticity, we could assume that these lines were the same for the chromatic channel. Upon the addition of the optimal contour width to the test line, the total width became 3.1–4.3 min (9.7–7.0 cpd). This line width could be detected by the chromatic channel. Despite this assumed integration of the test line and contour, color appearance of the test line and contour was reddish and white for cyan inducer. This appearance was confirmed by all six participants (including three naïve) in an additional query. The illusional perception of the colored test line contained coarse chromatic information with line widths of 3.1–4.3 min and detailed luminance information with test line widths of 0.9 min. This process may be a type of color capture by luminance signals.

### Optical Artifacts

To evaluate the influence of optical artifacts, we compared the matching data to the estimated artifacts using the proposed model (Marimont and Wandell, 1994). The estimated artifacts were as follows.

(1) Large artifacts on the dark test line: The aberration leaked a constant amount of visible light from the inducer and from the contour toward the test line, regardless of the luminance levels of the test line. Thus, the chromatic shift due to the artifacts on the dark test line was larger than that on the pale test line. The maximum artifact on the dark test line was 1.49, 0.96, 2.66, and 1.55 times larger than that of the pale test line for the cyan, red, violet, and yellow inducer, respectively. The artifacts increased as the luminance of the test line decreased, with the exception of the red inducer. The similar artifacts of the red inducer can be attributed to the longitudinal chromatic aberration, which induces a bluish color and cancels red light from the inducer [see (3) below].

(2) Large artifacts on thin lines: The artifacts were significant in the vicinity of the inducer and contour; thus, the chromatic shift due to the artifacts was large on the thin lines. This was confirmed for all the chromaticities of the inducer with respect to the pale test line. However, for the dark test line, the maximum artifact was the test line with a width of 3.7 min for the violet and yellow inducers. This can be attributed to the balance between the monochromatic aberration and longitudinal chromatic aberration.

(3) Bluish chromatic shifts: The chromaticity of the test line shifted toward the inducer chromaticity for the cyan and violet inducers but shifted away from the inducer chromaticity for the red and yellow inducers. The longitudinal chromatic aberration led to the selective dispersion of short-wavelength light from the inducer and the contour to the test line. The short-wavelength light corresponds to a shift in chromaticity toward cyan and violet. Thus, the chromatic aberration caused a chromatic shift toward the inducer chromaticity for the cyan and violet inducers and a shift away from the inducers for the red and yellow inducers. It should be noted that the magnitude of the chromatic shift was calculated by the shift along the corresponding cardinal axis and ignored the shift along the other axes, although the shift occurred in the three-dimensional cone excitation space. In contrast, monochromatic aberrations involve the simple scattering of the inducer chromaticity and usually cause a shift similar to that of chromatic assimilation. By integrating monochromatic and chromatic aberrations, the chromatic shifts would be large for the cyan and violet inducers, and the chromatic shifts would be small for the red and yellow inducers. The simulation revealed that the estimated artifacts for the cyan and violet inducers were large and shifted toward the inducer chromaticity for both luminance levels. For the red and yellow inducers, the estimated artifacts generally shifted toward the inducer chromaticity and away from the inducer chromaticity for several lines with thick contours. The thick contours prevented spreading from the inducers; thus, the artificial chromatic shift primarily reflected the longitudinal chromatic aberration from the contour, which corresponded to a chromatic shift away from the chromaticity of the red and yellow inducers.

It is unclear whether the parameters used in the simulations were consistent with those of the participants in the experiment. There were large individual differences in the opacity of the ocular optics and pupil diameters, and these differences influenced the balance between blurring and chromatic aberrations. The simulation results serve as estimations of the average chromatic shift across observers. Thus, future work is required to examine the relationship between individual differences in ocular optics and color matching.

In summary, the artifacts were found to be dependent on the line widths, luminance of the test line, and inducer chromaticity. The artifacts robustly shifted toward or away from the inducer chromaticity, depending on the inducer chromaticity. The direction of the artifacts was determined by the balance between monochromatic and longitudinal chromatic aberrations. The artifact of the pale test line was adequately large and shifted toward the inducer chromaticity for all the inducers; this shift was opposite to the perceptual color shifts. This indicates that the magnitude of the illusory effect exceeded that of the artifact, and the neural mechanisms predominate the illusory effect. For the dark-test line, the artifact was more significant, and it occasionally exceeded the magnitude of the illusory effect, given that less light of the test line was susceptible to the artifact.

### Luminance Dependency With Respect to Artifacts

Cerda-Company et al. (2018) found that luminance dependency switched depending on the chromaticity of the proximal surroundings of the Monnier–Shevell illusion. The color shift was large when the test ring was dark and when the proximal chromaticity was violet (S+). Conversely, the color shift was large when the test ring was bright and when the proximal chromaticity was yellow (S-). The effect of optical artifacts was not investigated experimentally using artificial pupil (Monnier and Shevell, 2003) or theoretically by simulations. The luminance dependency was qualitatively consistent with the effect of optical artifacts. We found that the estimated artifact was similar to the illusional effect when the line was dark and when the distant surroundings were yellow (corresponding to S+ proximal).

We previously reported that, with a decrease in the luminance of the test line, the illusionary effect increased (Kanematsu and Koida, 2020). The illusory effect cannot be sufficiently explained by chromatic aberrations, as observers consistently perceived the illusory color, irrespective of its fixation near or far from the stimuli that induced different chromatic fringes by chromatic aberrations. However, in the previous study, we did not quantitatively examine the effect of optical artifacts. Thus, the influence of the artifacts on the color shift for different luminance levels of the test line is uncertain.

**FIGURE 6:**
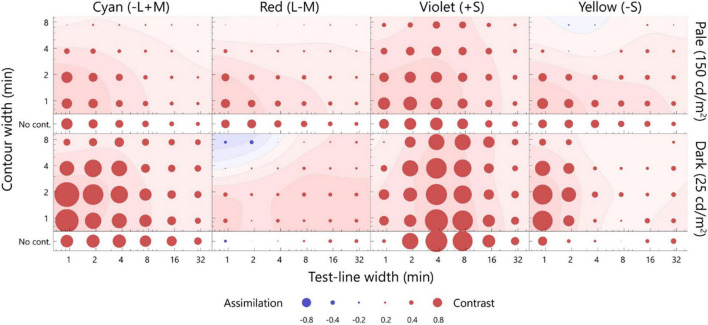
The difference between the matching and artifact. The difference was estimated by subtracting the artifact from the matching on the MB chromaticity, and considered as the neural factor of the chromatic induction. The upper row presents the pale test line, and the lower row presents the dark test line. The smoothed contour plots were plotted using the same calculation as that employed for Figure 4. The format is the same as that of Figure 4.

The results of this study revealed that the optimal widths and direction of the color shift were the same for the pale and dark test lines with respect to the cyan and red inducers, whereas they varied significantly for the yellow and violet inducers. The simulation results revealed that the artifacts led to a larger chromatic shift for the dark test line than the pale test line; thus, the observed change in the optimal width of the illusion between luminance levels may be due to the artifacts. To evaluate the influence of artifacts on the optimal widths, the following analysis was performed. In this analysis, we assumed that the observed matching was the result of a linear summation of the artifacts and neural shift. If this assumption is true, the neural shift could be plotted by subtracting the artifacts from the matching. The subtraction is shown in Figure 6. Generally, the contrast effect was observed for all chromaticities, and the color shift between the luminance levels was more consistent than that shown in Figures 4, 5. To compare the color shifts quantitatively, correlation coefficients were calculated for the pale and dark test lines (*r* = 0.93, *p* < 0.05, cyan; *r* = 0.63, *p* < 0.05, red; *r* = 0.63, *p* < 0.05, violet; *r* = 0.57, *p* < 0.05, yellow). They were all significantly and positively correlated; thus, the neural spatial properties were consistent across luminance levels. We therefore concluded that the changes in the optimal widths between the luminance levels for the S-cone chromaticities were mainly due to the artifacts and that the underlying neural mechanisms were the same, regardless of the luminance levels.

## Conclusion

The optimal width for the simultaneous chromatic contrast effect of the patterned surroundings differed between the cardinal axes. The optimal widths of the center line and contour for the L/M-cone chromaticities were 0.9 min and 1.1–1.7 min (as small as one cone photoreceptor), respectively, and those for the S-cone chromaticities were 8.2–17.5 min and 0.9–2.5 min, respectively. There is a high probability that these differences reflect the spatial resolution limit between the L/M-cone and S-cone chromatic modulations. The optical artifact influenced the chromatic shift, particularly for the darker test line; however, the chromatic contrast effect for the L/M-cone chromaticities (especially cyan) exceeded that of the artifact. Thus, it was concluded that the dominant factor of the color shift is neural rather than optical. The optimal line width for the L/M-cone chromaticities was finer than the resolution limit of the chromatic channel, thus indicating that the color appearance of the thin line is inferred by integrating broader chromatic contrast information and detailed luminance contrast information. This visual inference of color is particularly important for recognizing the color of distant and fine objects.

## Data Availability Statement

The raw data supporting the conclusions of this article will be made available by the authors, without undue reservation.

## Ethics Statement

The studies involving human participants were reviewed and approved by the Committee for Human Research at Toyohashi University of Technology. The patients/participants provided their written informed consent to participate in this study.

## Author Contributions

TK conceived, designed, performed the experiments, analyzed the data, drafted, and wrote the manuscript. KK supervised the study, experiments, and analyses, and drafted the manuscript. Both authors approved the submitted version.

## Conflict of Interest

The authors declare that the research was conducted in the absence of any commercial or financial relationships that could be construed as a potential conflict of interest.

## Publisher’s Note

All claims expressed in this article are solely those of the authors and do not necessarily represent those of their affiliated organizations, or those of the publisher, the editors and the reviewers. Any product that may be evaluated in this article, or claim that may be made by its manufacturer, is not guaranteed or endorsed by the publisher.
