# Supplementary material for: Influence of Stimulus Size on Simultaneous Chromatic Induction
Source: Front Psychol. 2022 Jan 24;13:818149. doi: 10.3389/fpsyg.2022.818149 (PMC8818722; doi:10.3389/fpsyg.2022.818149)
Supplement: Supplementary file 1 [file Data_Sheet_1.docx]

Supplementary Material

# N/A responses

The participants were allowed to select the question mark if no suitable color existed on the palette or if they could not distinguish the color of the line (hereafter N/A response). Fig. S1 shows the number of N/A responses for all conditions. The maximum number of N/A responses was 2 (9.5% for the total responses; 21) for the dark-test line surrounded by a violet inducer. The N/A responses were frequently observed for the thin- and dark-test lines.


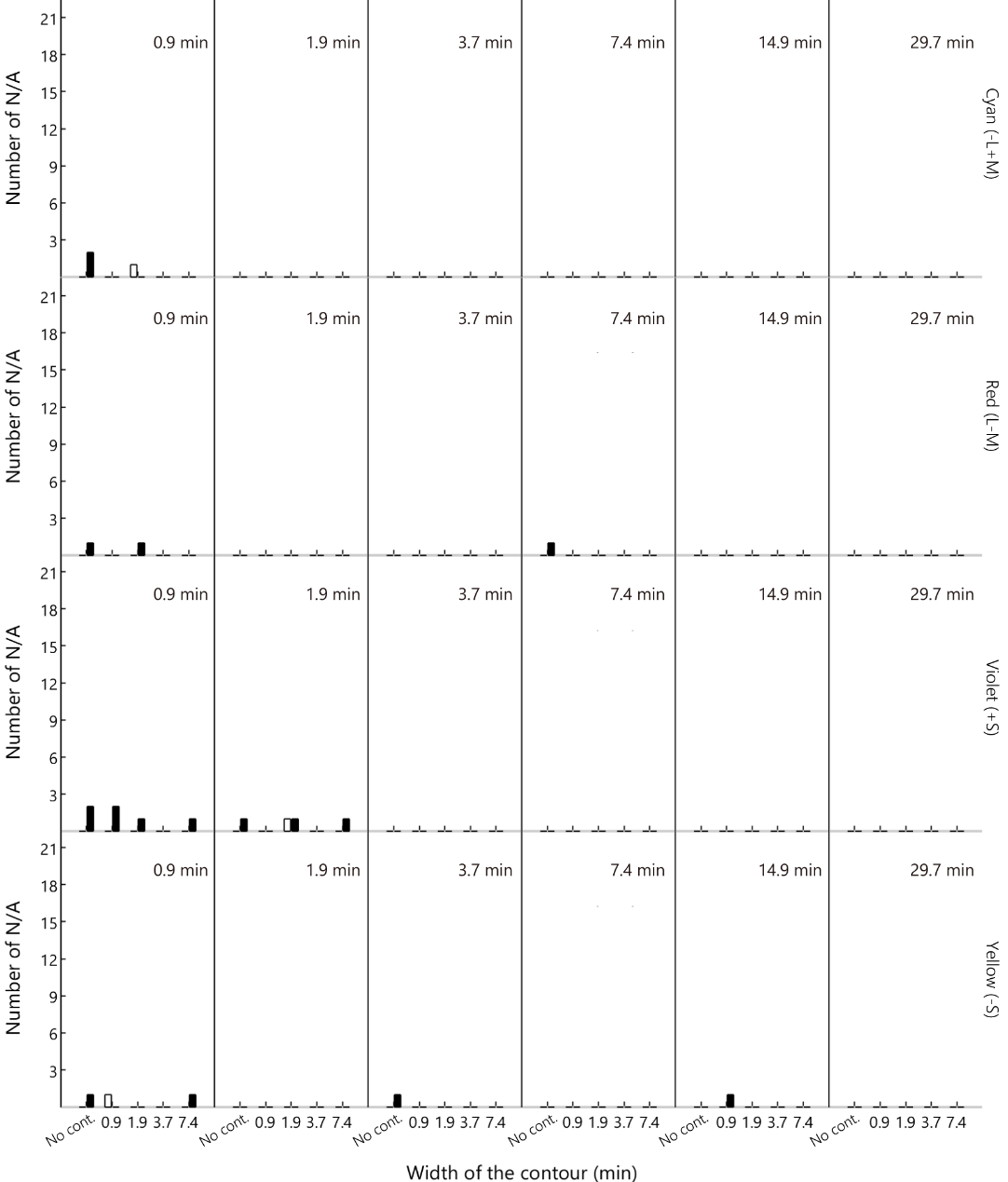


**Figure S1.** The number of N/A responses. Panels are separated by the width of the test line from left to right (denoted in the upper right corner) and the color of the inducers from top to bottom (cyan, red, violet, and yellow). The horizontal axis indicates the width of the contour, and the vertical axis indicates the number of N/A responses. The white and black bars indicate the response for the pale- and dark-test lines, respectively.

# Supplemental results

The matching and estimated artifacts are shown by the bubble plot in the main text. However, it is difficult to determine the specific value and variance of the bubble plot. Here, the data were plotted by the points for each width of the test line separately (Fig. S2, S3).


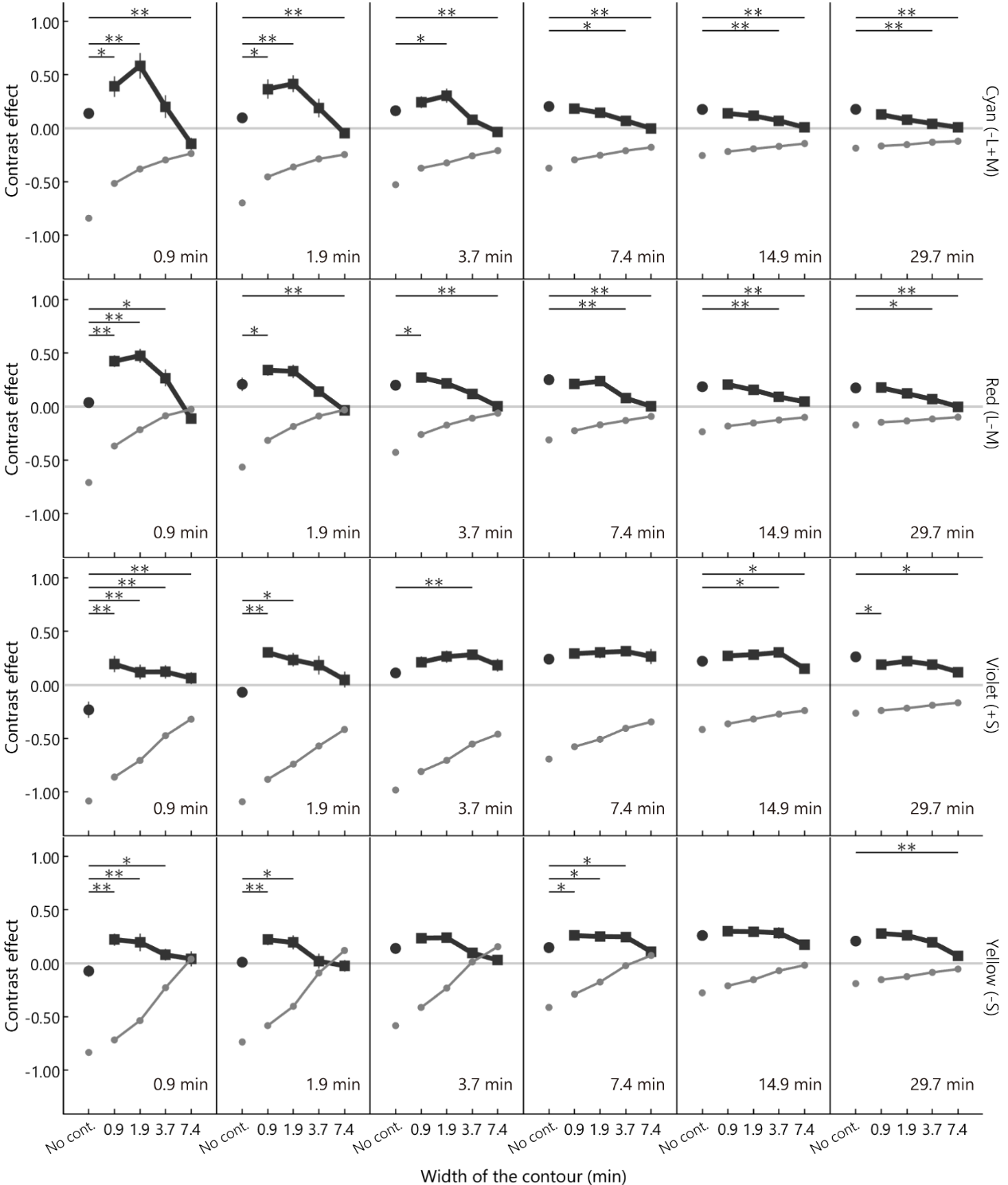


**Figure S2.** The matching and the estimated artifact of the pale-test line. The horizontal axis of each panel indicates the contour width, which corresponds to the vertical axis in Fig. 4 in the main text. The vertical axis indicates the contrast effect. The panels from left to right indicate the width of the test line. The thick square and circle indicate the matching of the contour and no-contour condition, respectively. Small gray dots indicate the estimated artifact. The error bar is the standard error of the mean. The asterisks indicate significant differences from the no-contour condition (**: p < 0.01, * : p < 0.05, sign test).


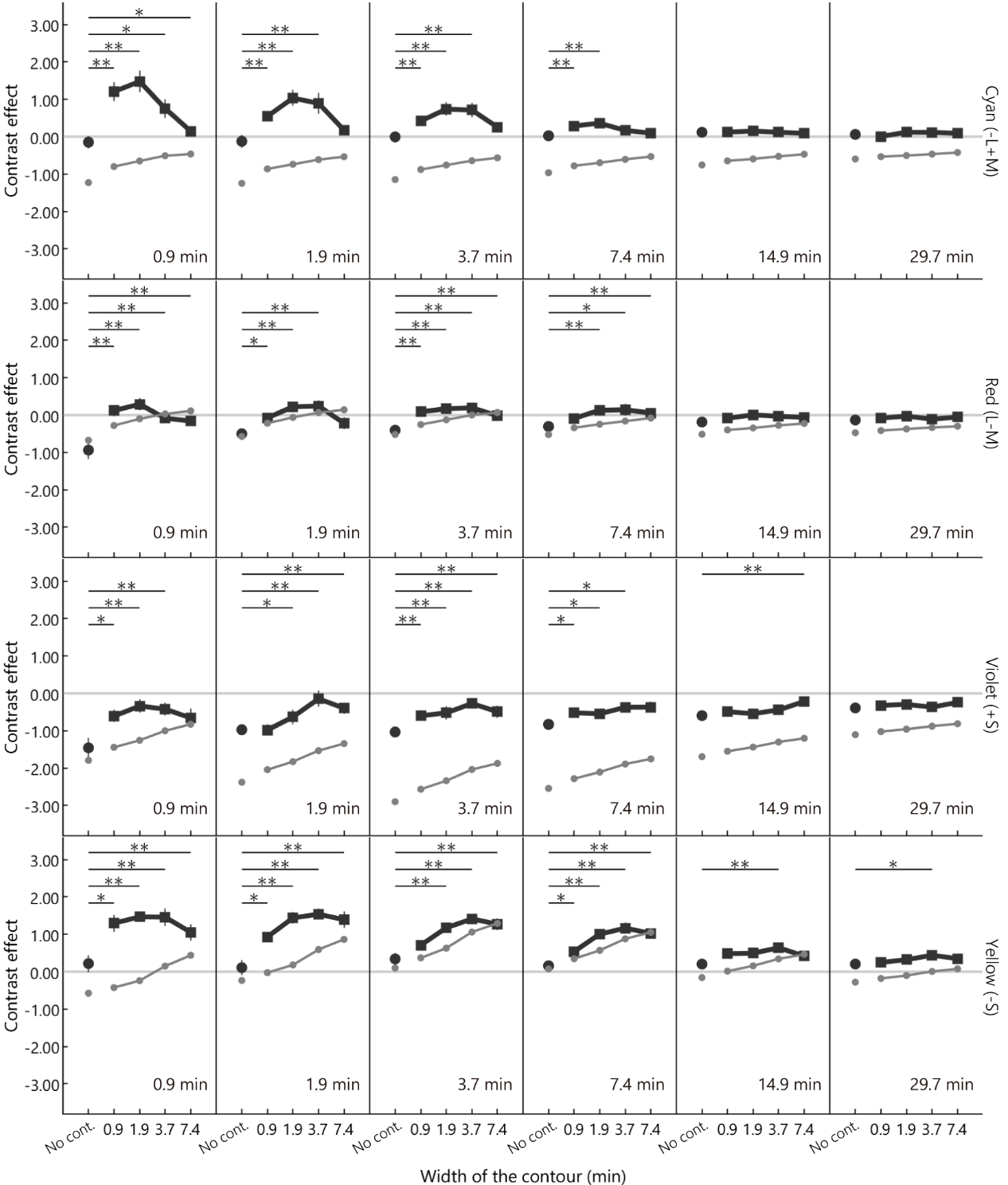


**Figure S3.** The matching and estimated artifact of the dark-test line. The format is the same as that in Fig. S2**.**

# Statistics

Analysis of variance was performed on the within-subjects factor (ranova function, Statistics and Machine Learning Toolbox version 12.0, MATLAB R2020b, Mathworks). The complete results are shown in Tables S1-S4.

**Table S1.** Two way repeated measures ANOVA of Contour condition for the pale-test line (150 cd/m2)

| **Inducer** | **Source** | **SS** | **df** | **MS** | **F** | **p** |
| --- | --- | --- | --- | --- | --- | --- |
| Cyan | Test-line width | 2.58 | 5 | 0.52 | 6.87 | 0.000 |
|  | Contour width | 7.30 | 3 | 2.43 | 34.44 | 0.000 |
|  | Test-line width x Contour width | 3.30 | 15 | 0.22 | 7.25 | 0.000 |
|  | Error(Test-line width) | 7.15 | 95 | 0.08 |  |  |
|  | Error(Contour width) | 4.03 | 57 | 0.07 |  |  |
|  | Error(Test-line width x Contour width) | 8.64 | 285 | 0.03 |  |  |
| Violet | Test-line width | 1.25 | 5 | 0.25 | 5.24 | 0.000 |
|  | Contour width | 0.84 | 3 | 0.28 | 4.98 | 0.004 |
|  | Test-line width x Contour width | 0.50 | 15 | 0.03 | 1.06 | 0.395 |
|  | Error(Test-line width) | 4.52 | 95 | 0.05 |  |  |
|  | Error(Contour width) | 3.19 | 57 | 0.06 |  |  |
|  | Error(Test-line width x Contour width) | 9.04 | 285 | 0.03 |  |  |
| Red | Test-line width | 1.57 | 5 | 0.31 | 15.49 | 0.000 |
|  | Contour width | 6.82 | 3 | 2.27 | 35.12 | 0.000 |
|  | Test-line width x Contour width | 2.01 | 15 | 0.13 | 7.35 | 0.000 |
|  | Error(Test-line width) | 2.02 | 100 | 0.02 |  |  |
|  | Error(Contour width) | 3.88 | 60 | 0.06 |  |  |
|  | Error(Test-line width x Contour width) | 5.46 | 300 | 0.02 |  |  |
| Yellow | Test-line width | 1.31 | 5 | 0.26 | 3.11 | 0.012 |
|  | Contour width | 2.67 | 3 | 0.89 | 18.48 | 0.000 |
|  | Test-line width x Contour width | 0.44 | 15 | 0.03 | 1.06 | 0.391 |
|  | Error(Test-line width) | 8.01 | 95 | 0.08 |  |  |
|  | Error(Contour width) | 2.75 | 57 | 0.05 |  |  |
|  | Error(Test-line width x Contour width) | 7.93 | 285 | 0.03 |  |  |

SS: Sum of squares, df : degrees of freedom, MS: Mean squares

**Table S2.** One way repeated measures ANOVA of No-contour condition for the pale-testline (150 cd/m2)

| **Inducer** | **Source** | **SS** | **df** | **MS** | **F** | **p** |
| --- | --- | --- | --- | --- | --- | --- |
| Cyan | Test-line width | 0.14 | 5 | 0.03 | 1.80 | 0.119 |
|  | Error(Test-line width) | 1.61 | 100 | 0.02 |  |  |
| Violet | Test-line width | 4.20 | 5 | 0.84 | 18.49 | 0.000 |
|  | Error(Test-line width) | 4.54 | 100 | 0.05 |  |  |
| Red | Test-line width | 0.56 | 5 | 0.11 | 4.99 | 0.000 |
|  | Error(Test-line width) | 2.24 | 100 | 0.02 |  |  |
| Yellow | Test-line width | 1.62 | 5 | 0.32 | 8.77 | 0.000 |
|  | Error(Test-line width) | 3.69 | 100 | 0.04 |  |  |

SS: Sum of squares, df : degrees of freedom, MS: Mean squares

**Table S3.** Two way repeated measures ANOVA of Contour condition for the dark-test line (25 cd/m^2^)

| **Inducer** | **Source** | **SS** | **df** | **MS** | **F** | **p** |
| --- | --- | --- | --- | --- | --- | --- |
| Cyan | Test-line width | 45.40 | 5 | 9.08 | 17.16 | 0.000 |
|  | Contour width | 16.46 | 3 | 5.49 | 11.02 | 0.000 |
|  | Test-line width x Contour width | 18.78 | 15 | 1.25 | 5.43 | 0.000 |
|  | Error(Test-line width) | 52.92 | 100 | 0.53 |  |  |
|  | Error(Contour width) | 29.88 | 60 | 0.50 |  |  |
|  | Error(Test-line width x Contour width) | 69.15 | 300 | 0.23 |  |  |
| Violet | Test-line width | 2.64 | 5 | 0.53 | 1.47 | 0.208 |
|  | Contour width | 5.16 | 3 | 1.72 | 3.96 | 0.013 |
|  | Test-line width x Contour width | 7.47 | 15 | 0.50 | 1.79 | 0.036 |
|  | Error(Test-line width) | 28.66 | 80 | 0.36 |  |  |
|  | Error(Contour width) | 20.87 | 48 | 0.43 |  |  |
|  | Error(Test-line width x Contour width) | 66.74 | 240 | 0.28 |  |  |
| Red | Test-line width | 1.84 | 5 | 0.37 | 1.95 | 0.093 |
|  | Contour width | 3.06 | 3 | 1.02 | 7.10 | 0.000 |
|  | Test-line width x Contour width | 4.14 | 15 | 0.28 | 2.74 | 0.001 |
|  | Error(Test-line width) | 17.94 | 95 | 0.19 |  |  |
|  | Error(Contour width) | 8.17 | 57 | 0.14 |  |  |
|  | Error(Test-line width x Contour width) | 28.69 | 285 | 0.10 |  |  |
| Yellow | Test-line width | 66.26 | 5 | 13.25 | 27.35 | 0.000 |
|  | Contour width | 10.12 | 3 | 3.37 | 11.44 | 0.000 |
|  | Test-line width x Contour width | 6.84 | 15 | 0.46 | 1.83 | 0.030 |
|  | Error(Test-line width) | 43.61 | 90 | 0.48 |  |  |
|  | Error(Contour width) | 15.92 | 54 | 0.29 |  |  |
|  | Error(Test-line width x Contour width) | 67.16 | 270 | 0.25 |  |  |

SS: Sum of squares, df : degrees of freedom, MS: Mean squares

**Table S4.** One way repeated measures ANOVA of No-contour condition for the dark-test line (25 cd/m2)

| **Inducer** | **Source** | **SS** | **df** | **MS** | **F** | **p** |
| --- | --- | --- | --- | --- | --- | --- |
| Cyan | Test-line width | 1.01 | 5 | 0.20 | 1.32 | 0.263 |
|  | Error(Test-line width) | 13.77 | 90 | 0.15 |  |  |
| Violet | Test-line width | 13.59 | 5 | 2.72 | 8.59 | 0.000 |
|  | Error(Test-line width) | 28.48 | 90 | 0.32 |  |  |
| Red | Test-line width | 5.24 | 5 | 1.05 | 6.05 | 0.000 |
|  | Error(Test-line width) | 15.59 | 90 | 0.17 |  |  |
| Yellow | Test-line width | 0.68 | 5 | 0.14 | 0.41 | 0.842 |
|  | Error(Test-line width) | 31.85 | 95 | 0.34 |  |  |

SS: Sum of squares, df : degrees of freedom, MS: Mean squares
